# FinTech: A New Hedge for a Financial Re-intermediation. Strategy and Risk Perspectives

**DOI:** 10.3389/frai.2020.00063

**Published:** 2020-10-15

**Authors:** Anna Omarini

**Affiliations:** Department of Finance, Bocconi University, Milan, Italy

**Keywords:** FinTech, open banking, platform, ecosystem, APIs, digitalization, re-intermediation, bank business model

## Abstract

The emergence of new technologies and players, along with a favorable regulatory framework (PSD2 Directive), is changing the banking industry. FinTechs and TechFins have allowed the introduction of new services and changed the way customers interact to satisfy their financial needs. The FinTech landscape is constantly evolving in the market. Different business value propositions are entering the financial services industry, moving from increasing the user's experience to developing a time to market framework for banks to innovate products, processes, and channels, increasing the cost efficiency and looking for a “partnering on order” to lighten the regulatory burdens for banks. The many businesses of banks are changing their value chains, and banks' business models should do the same accordingly. Strategists could no longer take their value chains as a given; choices have to be made on what needs to be protected and maintained, what abandoned and the new on coming to make banks evolve and become more resilient in doing their job. Banking is shifting significantly from a pipeline, vertical paradigm, to open banking business models where open innovation, modularity, and ecosystem-based bank's business model may become the ongoing mainstream and paradigm to follow and develop. Opportunities and threats for banks are many and new ones to re-gaining their role in the market throughout a re-intermediation process.

## Introduction

The rise of the ever-increasing relation between technology and financial services is bringing significant change to the banking industry. Shifting market conditions, customer needs, the entrance of new players, and digital technologies, along with new regulations—such as the Payment Service Directive 2 (PSD2) in Europe that aims to increase innovation, competition, and transparency—are all reshaping the banking industry and the financial intermediation model as well (Brueggemann, 2017).

There are many definitions of FinTech (Omarini, 2019, p. 198); however, it can be summarized that one main feature regards any technology that may reduce or eliminate the costs of financial intermediation especially in three broad areas of finance (Das, 2019, p. 981): (a) raising capital, (b) allocating capital, and (c) transferring capital.

FinTechs seem to be disrupting all the banks' primary functions of maturity transformation (through competition in lending), allocation (through robo-advisor and crowd investing platforms), payment services (through the introduction of new payment platforms and interfaces), and information processing (through the use of big data, machine learning, and artificial intelligence—AI), as well as liquidity provision and risk pooling.

The Financial Stability Board (Financial Stability Board – FSB, 2017) describes well that FinTechs enable financial innovation so that it could result “in new business models, applications, processes, or products with an associated material effect on financial markets and institutions and the provision of financial services.” Since FinTechs started entering the market, they have worked on two important selling points:

- Better use of data and
- Frictionless customer experience (speed of sign-up, no-fee transparency, peace of mind through activity notification, rich in choice, etc.) to deepen relationships

They have brought to the traditional banking industry a wave of competition and broken pipeline value chains, unbundling them into different modules of products or services, which may be combined among themselves. These companies on the one hand and the BigTechs (Google, Facebook, Apple, Samsung, Alibaba, etc.) on the other have been forcing the industry to change, transform, and evolve in a set of new financial intermediation directions.

Use of data and customer experience are both FinTechs' major assets and threats as well. On the one hand, they please the customers as individuals and introduce the paradigm of contextual banking. On the other, the two selling points are threatening both the incumbent players and regulators in different ways. For banks, it is even more urgent to react actively because their “no fee zone” is expanding, due to new regulations from the Consumer Financial Protection Bureaus (CFPB) and similar entities in different countries.

Advances in digitization “are increasing opportunities to create new products and services and transform businesses.” The competitive landscape is experiencing major discontinuities, such as “ubiquitous connectivity, industry deregulation, technology convergence. All this is blurring industry boundaries and product definitions. These discontinuities are releasing worldwide flows of information, capital, product, and ideas, allowing non-traditional competitors to upend the status quo. At the same time, competition is intensifying, and profit margins are shrinking (KPMG, 2016). Managers can no longer focus solely on costs, product and process quality, speed and efficiency. For profitable growth, managers must also strive for new sources of innovation and creativity. Thus, the paradox of the twenty-first century economy: consumers have more choices that yield less satisfaction. Top management has more strategic options that yield less value” (Prahalad and Ramaswamy, 2004, p. 1–2).

The industry is also experiencing new risks (data privacy, cyber risks, data protection, etc.) and a changing framework of some old ones (operational—because of cloud compliance features—reputational risks, etc.). Different geographies are developing specific regulatory frameworks, and this is going to impact the way and the degree to which the industry is becoming most adaptable to change. In particular, “the strength and nature of the competitive advantages created by advances in AI could also harm the operations of efficient and competitive markets if consumers' ability to make informed decisions is constrained by high concentrations amongst market providers. Some analysts caution that the path of AI-based financial services technology may be similar to the path of other technology-based platforms (De Reuver et al., 2017) that have trended toward high-levels of market concentration (e.g., in Internet search and messaging). An AI/machine learning performance model improves through an abundance of data. Models that have a large market presence, therefore, have a built-in self-reinforcing advantage as their gains in market share improve the performance model, which could in turn further their gain in market share” [Department of the Treasury (The U.S.), 2018, p. 57].

The article is developed as follows: paragraph 2 and related 2.1 look at understanding how FinTechs are impacting value chains in the financial industry; paragraph 3 outlines open banking, platforms, and ecosystems as the main paradigms for banking and banks. Paragraph 4 describes the oncoming framework of risks. Paragraph 5 develops a brief conclusion and develops a discussion on the true next challenges for each actor of the market. Under these circumstances, having vision and build strategies, business models, and organizations is fundamental to standing the test of time.

## FinTechs and the Value Chains in the Financial Industry

It is beneficial to remember how things worked before and after FinTechs and TechFins or big techs in the financial industry.

Banking models are shifting significantly from a pipeline, vertical, paradigm, to modular solutions that pave the way to new banking paradigms that entail higher levels of openness toward third parties and a growing number of modular services bundled together.

Value is created in platforms through economies of scope in production and innovation (Gawer, 2014). In order for platforms to work, adoption and network effects are essential. Models can go to mere compliance with the prescriptions of openness of PSD2, to the inclusion of new services, the opening of the banking core and data, and the aggregation of those within a platform experience. In particular, we assist both to the evolution of a Bank-as-a-Platform model and a tech-platform-driven model supporting banking and financial intermediation, which both constitute a new interesting field of analysis.

Since the wave of digital transformation started entering the financial industry, banking-as-a-business has started moving from a product/service perspective to more contextual solutions where providers are customer needs-driven. This is because customer-driven companies outperform the shareholder-driven ones, and this requires an outside-in approach.

Having said that, it is beneficial to remember that digital transformation implies four main categories of innovation (product, process, organizational and business model) (Omarini, 2019, p. 340); all of them require rediscovering that a new strategy paradigm exists. This regards the concept of co-creation, and because of this no single firm can unilaterally carry out a process of continuous experimentation, risk reduction, time compression, and minimizing investment while maximizing market impact. Co-creation requires access to resources from extended networks (suppliers, partners, and consumer communities).

Under these new market conditions, FinTechs have become an important piece of a bigger puzzle, each one in its own area of business (payment, lending, etc.), while at the beginning most of them started as mono-business companies. Only a few of them may become leaders in the market. On the one hand, there are those that make their strategy become international, and on the other, there are FinTechs which enlarge their services-scopes. However, the majority of them will become part of ecosystems where the direction could swing from banks to tech companies or to FinTechs as well, able to manage the network by developing kinds of conglomerate-as-a-service.

Another interesting point to outline regards this recent period where all of us have experienced lockdowns around the world, and some effects have also impacted FinTechs as well. The valuations of most unicorns have crashed overnight, while on the FinTechs side there are different situations. Some of them have experienced a dramatic reduction in their evaluation, others were quite lucky and suffered less.

There are many and different feelings on the way FinTechs will exit this situation, which as far as we understand has overall accelerated some strategic choices.

First of all, there are many and different FinTechs in the market. What is critical is to look at the fundamentals of the business. All of them are about answering what society is going to look like in the future (attitudes, behaviors, habits, etc.), so that if we no longer need to go to retail stores anymore, why do we need some services based on this situation? This, again, underlines that banking is a people business (Omarini, 2015) and this requires a business to be resilient to become adaptive to consumer changes or moves into a different market where you can still apply the service because the society is not yet ready to shift somewhere else, which means the same business in different markets. Just think of the ongoing situation where the recent wave of people is rethinking and restructuring their finances, so that they have decided to switch rates to digital banks. In this scenario, the winners are those that have enough liquidity—or better still cash-rich—to buy good technology and invest in new directions, also taking the opportunity to use the pandemic to its advantage. This is especially true for payments that are going to be increasingly contactless. However, some more lessons can be learnt from difficult times especially due to external factors such as the following:

- People costs and per-customer contribution margin are key factors, and valuable indicators. They are valuable for incumbents too. When staff costs rise, then this becomes a burden if growth is not going to move on. Then, if we move on the per-customer contribution margin (revenue, minus variable costs including credit losses), then this makes a FinTech earn more money per bank account than the cost of running those bank accounts.
- One more point has to do with the way a FinTech makes its revenues per customer, and net income is the figure to look out for here. This means that the more sources of revenues a company holds, the better it is for it. If we think of some of the best-known FinTechs, they gather their net income from interchange fees, ATM withdrawals, which can diminish during the pandemic, but gathering revenues from other sources such as lending, investing, or again from referring customers to third-party services, and earning commissions from these referrals.

Under this oncoming market structure configuration, a focus on control and ownership of resources is giving way to the importance of accessing and leveraging resources through unique ways of collaboration. “The co-creation process also challenges the assumption that only the firm's aspirations matter. (…) Every participant in the experience network collaborates in value creation and competes in value extraction. This result in constant tension in the strategy development process, especially when the various units and individuals in the network must collectively execute that strategy. The key issue is this: balancing act between collaborating and competing is delicate and crucial” (Prahalad and Ramaswamy, 2004, p. 197).

If co-creation is fundamental to the industry, this needs to leverage on a wider customer perspective that requires introducing the idea of developing ecosystems where the customer is truly free to move and choose the best deal in more competitive markets able to let consumers' ability to make informed decisions against any possible market concentrations among market providers.

A business ecosystem (Moore, 1996) reflects the new paradigm of competition in a better way. Traditional management models aimed at gaining competitive advantage, such as vertical or horizontal integration, economies of scale and scope, are not effective anymore. The value of today's companies is determined by the size of its ecosystem (Tewari, 2014). Business ecosystems consist in crossovers of a variety of industries, of which companies cooperate and embrace open innovation to satisfy new customers' needs and develop new products and services, to improve the customers' experience (Moore, 1996).

Finally, it is worth outlining that in order to increase efficiency and costs optimization, there has been an increase in the use of the cloud that has also been fundamental for FinTechs to take off. Cloud technology—a part of the new construct of software-as-a-service, SaaS—is enabling organizations across the economy to more rapidly innovate (Chesbrough, 2003, 2006, 2011; Chesbrough et al., 2014) by reducing barriers to entry and acquire high-quality computing resources. On the one hand, cloud computing enables more convenient, on-demand access to computing resources (e.g., networks, servers, storage, applications, and services) [National Institute of Standards and Technology (NIST), 2011]. On the other, it makes banks and other financial service providers rely increasingly on third-party providers by increasing some related concerns and risks.

### The Nascent Business Ecosystem: Concept, Rationale, and Approaches of Analysis

Ecosystems are cross-industry entities (Moore, 1993) where there is a loose of the “networks of suppliers, distributors, outsourcing firms, makers of related products or services, technology providers, and a host of other organizations that affect, and are affected by, the creation and delivery of a company's own offerings” (Iansiti and Levien, 2004b).

They are sets of data and features that combine to create value. The ecosystem economy is therefore linked to the marketability of the information that can be produced thanks to the integrated management of its data.

Ecosystems are characterized by both symbiotic and antagonistic relationships, without which each single player would lose its own individual meaning, so that the value relies on the interdependencies among actors (Adner and Kapoor, 2010; Gawer, 2014; Gawer and Cusumano, 2014). While the boundaries of an ecosystem may be blurred, companies should try to identify the players on which their success depends. In doing this, a new intermediation model is emerging, where different players can take several roles such as the “keystone” (Iansiti and Levien, 2004a and what Moore, 1993—initially defined as “leader” or “focal firm” according to Adner and Kapoor, 2010). This is a firm that furnishes a set of common resources on which other players can leverage.

Trying to make a parallelism, the ecosystems in which banks could find themselves working in require banks to look for a new re-intermediation model. This is because ecosystems are a technology stack structure supporting different value propositions which are mediated by the presence of other participants that increase “system value through direct and indirect network externalities” (Parker et al., 2016). In the meantime, this also “increases the likelihood of serendipitous interactions between partners, which may unlock new interactions and combinations” (Parker et al., 2016). In the ecosystem, partners have to focus on reaching a threshold level of coordination and create the (endogenous) boundary of the relevant ecosystem. The coordination is the key issue of a business ecosystem that under digital transformation is increasing its dependence on digital premises (Pagani, 2013). These partners may be, among others, the FinTechs that from an initial wave of fragmentation of the financial industry are now becoming the pillars of it by offering and increasing modularity and distributed banking throughout the re-bundling of their and others' value propositions. It is also worth outlining that in this scenario, financial services behave as a strong catalyst for the nascent ecosystems. This in fact allowed a major integration among interdependent, yet distinct modules belonging to the three areas of finance (raising, allocating, and transferring capital).

This takes us back, *mutatis mutandis*, to the main reasons why banks exist in the market (transaction costs and the problem of imperfect information, market signaling) (Benston et al., 1976; Leland and Pile, 1977; Campbell and Kracaw, 1980; Fama, 1980; Diamond and Dybvig, 1983; Diamond, 1984). In a nutshell, this is because they are information specialists and liquidity providers and are also able to transform and accept risks.

While the core objectives of financial intermediation have remained the same, the methods and functionalities relating to those objectives have been changed by new technology and market developments. At present, data analytics is frequently the preferred method of choice, and automated online computer programs are the favored functionalities of choice. Automated, algorithmic computer programs are now at the forefront of financial innovation. Just think of some of the human-led efforts in finance that have been replaced by artificial intelligent programs.

All this focuses attention on two points of analysis worth outlining. The first one is that like its traditional counterpart, new financial intermediation looks at developing the core purposes of financial intermediation, albeit by introducing new methods and functionalities (Lin et al., 2015). The second point is that (Brainard, 2017, p. 3): “More often than not, there is a banking organization somewhere in the FinTech stack. Just as third-party app developers rely on smartphone sensors, processors, and interfaces, FinTech developers need banks somewhere in the stack for such things as: (a) access to consumer deposits or related account data, (b) access to payment systems, (c) credit origination, or (d) compliance management. For instance, account comparison services rely on access to data from consumers' bank accounts. Savings and investment apps analyze transactions data from bank accounts to understand how to optimize performance and manage the funds consumers hold in those accounts.” All this is due to the new ways (such as websites and apps) for intermediaries to interact with their clients.

Under these circumstances, we have to remember that financial services are fiduciary based, so that the more the ecosystem and its network are expanding, the more critical limitation of direct transactions may emerge in the market. “Taking this into consideration, there is the natural mutual distrust that derives from not knowing each other well. (…) All this requires the agents of the network to trust the network itself (so that) there is a need to reduce the trust gaps to benefit from new technologies in the presence of large trust gaps.” On the one hand, banks, again, “will produce and process the information needed to enable millions of anonymous individuals to interact and trade on the web, while their reputational capital and expertise will be necessary to validate the quality of the information exchanged.” On the other, “as networks bring in more participants and business opportunities, such knowledge will be useful for the intermediaries themselves to provide risk aggregation and diversification services that cannot be performed by individual agents or that may be too costly for individuals to perform” (Omarini, 2019, p. 18–19). All this seems a “win–win situation” for both incumbent banks and FinTechs.

Any further steps into the era of e-finance will make the circuit process look increasingly sophisticated, and in the meantime, it reaffirms the virtuality of bank money—based on the promised issued by specialized entities—and will always call on banks to give money a real content and preserve it.

These are the roots of the open banking paradigm, where money, production, and investment have to be considered in an integrated way, where banking and finance interrelate differently over the economic development, but performing complementary functions essential to the economy, leading to different efficiency/stability configurations, which are the next challenges for regulators and authorities to foresee and discern.

## The Shifting Paradigms of Banking and Banks: Open Banking as the Game Changer of the Renewed Financial Industry

Ensuring a proper working of competitive market forces can be considered one of the main reasons for open banking (*alias* PSD2) in Europe and other countries, where the goal of promoting competition in financial services is an explicit component of the regulator's mandate (Deloitte, 2017). Its adoption is at varying stages in 35 markets relating to products that account for approximately 90 percent of revenue pools in those markets (McKinsey, 2019, p. 11, 12).

PSD2 can be described as “A legislative framework to facilitate the entry of (such) new players and ensure they provide secure and efficient payment services. (…) making it easier to shop online and enabling new services to enter the market to manage (their) bank accounts, for example to keep track of (their spending) on different accounts” (European Commission, 2015). With this, many competitive boundaries have started to loosen because of deregulation and the reduction of borders among industries so that banks have found themselves facing massive competition in many of their business areas (card payments, current accounts, consumer loans, some insurance products, financial planning, and family cash management). It is worth outlining that payment services are the entry gate for every other financial need (Omarini, 2019); transferring money is the most important pillar for any service extension. In fact, the “competition between banks and big techs is already fully visible in the area of payments where the market share of non-bank electronic payment providers, which offer alternatives to traditional credit and debit cards, is growing. Nearly 60% of retail banking transactions worldwide are now estimated to go through mobile and online providers, which offer alternatives to traditional credit and debit cards are growing” (Swiss Finance Council, 2020, p. 84).

Also, from the Basle-based BIS's annual Red Book report on payments and financial infrastructures, it is outlined that there are increasing incursions by non-bank competitors into both retail and wholesale payments, so that “The traditional bank-based ecosystem is being disrupted from below by FinTechs and from above by well-established big techs,” states the report. This means that a new framework of financial intermediation system may emerge from the combination of incumbents, FinTechs, and big techs.

Big techs provide banking-like and other financial services together with their feature of being intrinsically linked to the rise of big data and data analytics and their related opportunities. All this is becoming an important driver for changing the automated decision-making process based on technologies, like artificial intelligence, and therefore make some impacts on the financial intermediation model.

However, it is worth outlining that “there are jurisdictional differences: the penetration of big techs in payments is more prominent in countries where the use of other cashless means of payments (e.g., credit cards) is low. For instance, big tech mobile payment services account for 16% of GDP in China” (Swiss Finance Council, 2020, p. 87).

If payments act as the entry level for them into the financial services industry, some big tech firms are also active in lending and asset management. Again, “there are geographical differences. For instance, the provision of credit by big techs has expanded more strongly than other FinTech credit in those jurisdictions with lighter financial regulation and higher banking sector concentration. These lending services have mainly been developed to sustain big techs' e-commerce platforms, and the data derived from e-commerce transactions have become a powerful tool for big techs in providing loans to consumers” (Swiss Finance Council, 2020, p. 87). On the expansion of big techs into asset management, this is mainly driven by their payment platforms and often regards a set of short-term investments, such as money market funds from customers' accounts' balances.

Until now, the emergence of big techs has not led to the disintermediation of the banking system. They have often acted as distribution channels relying on existing infrastructures like bank accounts or correspondent banking for cross-border transactions. Another point to make regards the fact that big techs still depend on big banks to access customers' accounts and big banks can benefit from big techs' network effect to expand their customer base, this seems to reach a win–win game, so that partnerships between them might increase. Just think of the partnering of Apple with Goldman Sachs for credit card provision to name but one. They have also become useful partners to banks by providing big banks with technological infrastructures such as cloud computing for data storage and processing. Another link between the two players is that of funding. This occurs because big techs fund themselves from financial markets and financial institutions like banks.

As a matter of fact, we can see that banks and big techs are developing different frameworks of collaboration, which are having their momentum at present. However, competition between the two players may rise, and this comes from a future question which will regard to what extent big techs will eat into big banks' revenue share and profit margins. This may be possible because these firms have low-cost structures that can easily be scaled up—they were born to be platforms—and become able to provide basic financial services, especially delivering these set of services to the underbanked and unbanked segments of population.

Their competitive edge also comes from the fact that for regulatory and reputational reasons, banks have thus far not been as effective as big techs in harnessing data, and network externalities, and if things remain like today, big techs would not have to face high capital requirements, massive and complex regulations and stringent compliance (AML/KYC), and security (data, cyber) obligations. In the long-term horizon, big techs “by partnering with licensed banks can offer financial services to their customers without having to accept deposits and become subject to strict banking regulation. The best-known example of such a collaborative platform is to be found in payments with the widespread adoption of APIs. But other forms of partnerships between global banking and big techs are emerging in, for instance, bank loans to technology firms' customers such as small and medium-sized enterprises (SMEs)” (Swiss Finance Council, 2020, p. 91). All this is creating new scenarios in the financial service landscape where barriers are diminishing, and stability and customer trust are once again becoming important issues.

### Some Further Reflections on Open Banking

All the above is putting important roots for market regulators and market forces to boost the open banking (OB) agenda. As noted, UK regulators are taking a very active approach to open banking so that the Competitive Market Authority (CMA) has implemented its own reforms sometime beyond the PSD2. Further, the CMA has decided to set up the Open Banking Implementation Entity (OBIE) to support industry transformation. It is also interesting to outline the regulators' approach in China, where they developed an opposite framework by taking a more organic hands-off approach. That dichotomy shows that there is no single regulatory path or approach to open banking; local customs, standards, and expectations will dictate what is best. However, that is the direction, at present.

Incumbent banks understand that OB can assist with customer onboarding, retention, and satisfaction. As more FinTechs make their mark, there is an appetite for greater collaboration across the board. Banks are looking to improve customer value by adding some pieces of FinTech services to their existing financial expertise. This can be good, but it might not be enough to compete. This is because the greater focus on good customer outcomes means that services like categorization and aggregation will be table stakes. The winners will be the ones that place users at the heart of their approach and focus on delivering tangible customer value. Banking's holy grail is a combination of personability and relevance, and this is because this paradigm of banking will increase the number of conglomerate-as-a-platforms which are profitable and resilient only if they are able to develop themselves on a consistent and coherent customer experience evolutionary model. This evolution requires being rooted within a common framework of customer value and a strong innovative cultural organization that the entire conglomerate should outline in new rules for being an ecosystem where each part requires reliance on others' well-being.

From a technological point of view, open banking relies overall upon open Application Programming Interfaces (APIs) that are a set of codes and protocols that decide how different software components should interact. APIs are essentially allowing different applications to communicate with one another (Deutsche Bank Global Transaction Banking, 2018). APIs represent the interface through which third parties can develop and provide their services (*alias*, open innovation) defining the scope and level of access to the platforms (Microsoft Avanade and Accenture, 2017).

Through open banking, APIs are nowadays being used to issue commands to third-party providers. Before, they were used to connect developers to payment networks and display some details such as that of billings on a bank's website. They also allow for a close-to-seamless melding of services. In addition, transactional data unlocks a huge potential for greater transparency, and this also increases responsibility in the credit decision-making process.

Banks that are looking to out-pace their competitors are embedding new services into apps and websites, choosing to partner over doing it themselves. An increasing number of companies are realizing the impact a solid API strategy can have on their business, and banks are among them. This is because if 2018 signaled the huge potential presented by open banking, 2019 was the year OB started becoming realized on a more massive scale—for banks, businesses, and consumers alike. Under these circumstances, the challenge, at present, is that of balancing both endogenous and exogenous evolution.

APIs are also useful to develop Banking-as-a-Service (BaaS) to function properly; this is a key component of open banking (Zachariadis and Ozcan, 2017; Omarini, 2019).

BaaS is an end-to-end process that connects FinTechs and other third parties to banks' systems directly through the use of APIs. It helps to build up banks' offerings on top of financial providers' regulated infrastructure. However, a further step is Banking-as-a-Platform (BaaP), which is the next logical step that goes far beyond compliance with PSD2.

Banking-as-a-Platform represents just a subset of open banking, in which the choices of value and openness that banks make create several ties and roles with peculiar economics. BaaP builds on the advantages of open innovation, in putting together diverse know-how and resources (Zachariadis and Ozcan, 2017). Platforms are constructs that have the fundamental role of mediating relationships among different sides of users by reducing transaction costs and generating network effects.

They are organized around a core of elements that can constitute the basis for building innovative solutions and aggregating them toward a wider proposition. This emerging strategy acknowledges the modularization of banking services, but it tries to take advantage of the new opportunities that it spurs. Banking is indeed susceptible to migrating toward a platform model to pursue new revenue streams, as competition from FinTechs and TechFins might be unbeatable for a given set of services. The result would be an innovative proposition, supported by new business model frameworks, in which players share the costs of innovation and modules are aggregated to provide added-value services or bundles of services, and in which banks might forgo certain modules to concentrate in the orchestration of the network.

Banks, therefore, can take an active role in matching groups of users (e.g., FinTechs; developers; vendors; consumers, etc.), being the mediator through which all the groups get in contact with each other as well as become an orchestrator of the infrastructure. In doing so, they may regain their centrality in the economy and overall in their customers' everyday life. For many reasons (below zero interest rates, low profitability, increased new competition, value chains deconstructions, etc.), banks have to become an active player in a new re-intermediation open model where value is created in and through platforms and driven by nascent ecosystems business models.

In fact, open banking is an umbrella name to develop many new business frameworks over the next few years.

As a matter of fact, all the new constructs will be the frameworks for infinite interconnected financial intermediation ecosystems where banking is becoming an “enabler,” and under these circumstances banks may still retain some significant strengths in entire segments as well as resources—e.g., regulation expertise, licenses etc. (Deutsche Bank, 2014; Omarini, 2017). At this point, rather than merely providing a product, FinTechs can further act as an agile consultative partner in the implementation process. In these ways, they can help incumbents to tackle their technological and organizational transformation, while keeping up with—or getting ahead of—competition.

Finally, trying to summarize all the above, Open Banking acts differently according to the player party. On the one hand, if we look at FinTech this may be a kind of detonator to scale up and become profitable. On the other, if we look at the incumbents' side, then this is an opportunity to disrupt and make them evolve from both the inside and the outside.

However, not all the incumbents are looking at Open Banking as an opportunity to change. This is because there are some pros but also some cons. The latter has to do with the different culture, organization, and skills available in FinTechs compared to those belonging to incumbents.

Among the incumbents that are making the most from the new environment, we can outline BBVA, HSBC, and Goldman Sachs, just to mention a few of the more interesting examples. All of them are undertaking different strategies and related actions to overcome the new environment.

These examples show how important it is to look at OB as a way to improve and boost their core market but also look beyond it in order to increase their resilience and develop a strong strategy. A final point, Open Banking, is not only for big firms; it can be developed under a strong commitment such as the case of Banca Sella, a medium-size Italian banking group.

## Risks Framework in the Oncoming Scenarios

We are currently in the early stages of transforming the banking sector and the implementation of new technologies, where both regulators and supervisors have to face the additional challenge of the digital transformation, which requires achieving the right balance between promoting new digital value propositions—and protecting against the risks inherent to the digitalization of financial services (Gonzalez-Paramo, 2017).

In the above scenarios, there are old risks as well as new ones. The latter come from the increasing use of big data, robo-advisor platforms, AI, and machine learning and other seamless tools for tutoring customers, all of them aimed at increasing customer personalization and user experience to deepen relationships.

All this is increasing attention on both consumer protection and product governance regulation, because more innovation is contextualized in other customers' needs and it is fundamental to protect a true well-informed customer choice.

If we consider financial innovation in the context of consumer protection, it can be said that innovations “do not necessarily create new problems, but they have a tendency to aggravate the existing challenges of asymmetric information, market power imbalances and other imperfections that typically characterized markets for retail financial products” (Lumpkin, 2010, p. 39).

Another interesting point is that FinTechs are promoting a massive use of open APIs through mobile devices. On the one hand, this is rising the IT interdependencies between market players and infrastructures, and by this way IT risks increase IT risks events, which could escalate into a full-blown systemic crisis (Waupsh, 2017); new forms of moral hazard and shadow banking may come into the industry. On the other, smart, connected products tech-stack driven provide a gateway for data exchange between the product and the user and integrate data from different key points, such as business systems, external sources, and other related products. All this is increasing the customization and personalization of financial services because of the changing way of customers' interactions, where those relationships are becoming continuous and open-ended (Porter and Heppelmann, 2014). However, this may raise the lock-in effect for customers and possible sub-optimization in their decision processes and selected choices.

From a managerial point of view, this is also challenging functions and related processes requiring a far more intense coordination among old and new functions and skills able to manage new forms of cross-functional collaborations. Finally, this also forces companies to redefine their industries and rethink almost everything they do, by starting with their visions and strategies.

All this has a great potential to transform the banking industry significantly, and as a consequence, most regulators and supervisors around the world have taken a closer look at this situation also monitoring both opportunities and risks that technology may bring to the industry. This is because, on the one hand, the market is experiencing new ways of using data, new types of market players, and business models. On the other, there are also new cyber threats among the top issues for regulatory bodies to focus on (see Table 1). The regulatory response has happened at different speeds globally and in the next few years will shape the future of financial technology and the industry as well.

**Table 1:** FinTech: regulators' focus.

**Area of regulatory focus**	**Regulatori objectives**	**Regulatory response**
Data usage	- Protect individual privacy - Ensure data is not misuses or manipulated - Prevent data leakage - Prevent unethical use of data	- Data protection and data privacy requirements - Advice on ethical aspects of using data
New market players and business models	- Support competition and innovation - Set level playing field for FinTech firms and banks - Secure the safety of the financial system as a whole	- Opening client data to FinTech firms in a secure manner - Licensing and authorization of FinTech firms - “Same services, same rules” approach - Encouraging responsible innovation - Technology-neutral rules
New cyber threats	- Ensure cyber security and client protection	- Customer awareness - Secure communication - Strong customer authentication - Technical preventive measures - Fraud monitoring and detection

*Source: (Deutsche Bank Global Transaction Banking, 2018), p. 7*.

On this issue, it is worth outlining a recent choice made by the Australian authorities that has delayed the introduction of open banking rules overall because of testing and security of the new provisions for account data sharing. Under the new deadline (1 July 2020), consumers will be able to ask major banks to share their credit and debit card information, as well as deposit account and transaction account data with accredited service providers. A further step regards consumers' mortgage and personal loan data that will be shared after 1 November 2020.

In the new framework, consumer data right needs to have a robust privacy protection and information securities, and this requires establishing appropriate regulatory settings and IT infrastructure around the world.

As mentioned above, for regulators, there is also the issue of protecting customers from misconduct and reassuring them about making the right choice. In fact, there is a vast body of literature showing that consumers tend to make poor financial choices, such as not buying the “best value” products on offer and so taking on too much debt, misunderstanding investment risk, and choosing financial products that do not meet their real needs.

A big issue for regulators is also to keep up both stability and competition, which might become weaker, the more consumers' freedom decreases in the market. The main reason for this depends on some developed constrains to customer mobility as well as the trap of being so well-known that for the customer it is difficult to quit the situation. This moves the focus for regulators on the systemic risk from being “too-big-to-fail,” which refers to a few large financial intermediaries, to the systemic threat of “too linked to fail” (Lin et al., 2015), which includes instruments and intermediaries that are small in value and headcount but could destabilize the system because of the role they play in the networked marketplace.

It is worth outlining that most of the authorities and regulators have the approach of looking at FinTech as a single entity, on a case-by-case basis given the wide range of underlying applications (DTCC, 2017); other work is done on the definition of risks (BIS, 2017).

This can only be considered an initial step, because FinTechs are not going to remain that way for longer. They are becoming more and more part of the banking industry, and most of them are already partnering with banks or developing different frameworks of collaboration.

This wave of change finds its root in the way technological disruptions, along with regulation, could move an industry from a vertically integrated model to a multisided platform model. Therefore, moving attention from the single FinTech company to the platform framework might benefit the stability of the market as a whole, and the single company itself.

For a true competition in the ecosystems, regulators should consider the way the number and intensity of participants in the ecosystem are made possible. This fact shifts attention toward the level of openness of a platform, which is strictly linked to the need of alignment, coordination, and robustness of the platform itself, and the exact selection of the openness choices that may have to do with its stability and soundness. This factor may generate a trade-off between value capture (hindered by a too-open approach), and value proposition and platform adoption (hindered by a too-closed approach), which is a conflict between profiting from the platform and the network effects and reduction in costs it generates for all participants (West, 2003).

All this above increases systemic value, and it may produce some direct and indirect network externalities (Parker et al., 2016). This means that the resiliency of such business ecosystems requires having a threshold level of coordination to align each member in the overall value blueprint.

When it comes to the bank-specific managerial implications of such a platform choice, a bank might have to choose an approach in which it should hold restrictive terms and conditions and a burdensome due diligence process to ensure compliance with the law, of which the bank is ultimately responsible. This is because by widening the scope of the platform and augmenting the number of modules to sustain economies of scope and the related network effects, this could result in bottlenecks from ancillary activities or even lock in effects for customers. This requires a platform to compete effectively on each side to attract a fair number of members of each group not to create the incentive to subsidize those categories, which would generate most network effects for other parties, putting competitive pressure on prices (Armstrong, 2006), or/and decreasing transparency from a customer protection perspective.

In addition, the open approach toward banking may raise potential instability and risk factor, where the multiplication of the actors heightens the complexity of the system and creates potential breaches. Yet, it remains to be seen whether the technical standards and the due diligence conducted by banks will be sufficient in mitigating the risk of breaches and misuse of data and, more broadly, operational risks (large-scale theft, data corruption, etc.).

From the Basel agreements onward, the regulatory framework has changed the focus from what and how a bank can do to what a bank can do according to its capital adequacy by, first, mapping, and then managing the many risks it can undertake in doing its activity. Regulators have reinforced the prudential regulation compared to the structural regulation to reduce the risk of bank failure by prohibiting banks from getting involved in activities, which are judged by policymakers to be “too risky.”

In this approach, there are two possible weaknesses. The first one is that the set of prudential measures is affected by a strong endogeneity, which is the property of being influenced within a system. The second weakness is that regulation is trying to overcome a situation through tools based on a set of linear relationships, but overall, the oncoming financial intermediation system is neither linear nor simple. On this same issue, it is interesting to report the US Treasury's recommendations, which are the following (The US Department of the Treasury, 2018 p. 15):

- Adapting regulatory approaches to changes in the aggregation, sharing, and use of consumer financial data, and to support the development of key competitive technologies;
- Aligning the regulatory framework to combat unnecessary regulatory fragmentation, and account for new business models enabled by financial technologies;
- Updating activity-specific regulations across a range of products and services offered by non-bank financial institutions, many of which have become outdated in light of technological advances; and
- Advocating an approach to regulation that enables responsible experimentation in the financial sector, improves regulatory agility (…).”

## Discussion on the Main Conclusions

There are some important trends that have arisen in the market over the last decade such as the following:

- The nonbank sector has become powerful in the market so that regulatory challenges placed on traditional financial institutions have increased, such as those including the launch of numerous startup platforms;
- Most of these platforms have grown fast and beyond their startup phase. They have also implemented technology-driven approaches to onboard customers as well as process consumers' requests;
- Innovative new platforms in the nonbank financial sector are, in some cases, standalone providers. However, there are also others focusing to provide support for or interconnectivity with traditional financial institutions through partnerships, joint ventures, or other means;
- Big tech-driven companies holding a huge amount of consumer data have simultaneously entered the financial services industry, primarily in payments and credit provision;
- Over time technology-enabled competitors have scaled up, and the corresponding threat of disruption have raised the crossbar for the existing firms to boost their innovation processes in a faster manner and also look for dynamic and adaptive strategies. As a result, mature firms have launched platforms aimed at reclaiming market share through alternative delivery systems and at lower costs than they were previously able to provide.

This requires new strategic thinking which is moving on regarding the future of money that has become a more complex subject.

Money is the tool through which savings, investments, and capitals are held in the economy, no matter the form (digital or otherwise). Therefore, money changes follow changes in society. However, the minting of any representation of value is backed by the full faith and credit of the issuer whoever that may be.

At present, value is in the financial needs (spending, savings, lending, etc.) and in their related and different performances (monetary and not-monetary benefits).

The new current outlook reveals nascent ecosystems made of independent actors, where the traditional supply-centered oligopoly is coupled with FinTechs, TechFins, retailers, etc. Within this lies the disruptive aspect of PSD2 in Europe and similar trends in other markets. This is a key milestone itself in the unbundling and modularization—and more recently the re-bundling—of many and different banking and non-banking services which is challenging the financial services landscape (Omarini, 2019, p. 369).

The difference with the past on the relationship between technology and banking is the stronger interdependencies from a double perspective: technological and, even more important, strategic interdependence.

As mentioned above, the challenge for regulators is to move from a single-entity perspective (FinTech or bank focus) to a broader perspective, based on the banking conglomerate-as-a-platform. In particular, because platforms develop interactions among different, new and old, stakeholders, innovating fitting might require the development of new rules. In this, there is a critical point to control, which is the balancing of the power among the different actors. This is particularly true when banking and financial intermediation is increasing reliant on technology, and on the other way around, technology is driving the banking and financial businesses, pushing them sometimes outside the traditional boundaries. This may open the door to the next wave of shadow banking the more financial services are hidden in everyday life of customers and diluted in their habits. In this new changing game, consumer trust still remains a central component for each player in working toward open banking. From the financial institution through to third-party provider relationship and potential suppliers in between, there is a necessity to build and maintain consumer trust that will act as a catalyst for building competition. This trust requires both regulators' and companies' attention to third-party risks and relationships that have augmented for many different reasons, including those related to consumer's concerns, information security concerns, and other operational risks.

There are two main reasons why banks should react to this changing environment by actively managing their business lines. The first one regards the need for them to regain their centrality in their customers' everyday lives. The second reason is that banks are expected to react because of the low interest rate situation affecting bank intermediation margins.

At present, the response depends on whether or not the situation is perceived to be long-lasting by each bank in the market.

According to BIS (2019a, p. 3), “low interest rates encourage banks to rebalance their activities from interest-generating to fee-generating and trading business lines. The impact is economically significant. According to our estimates, the long-term elasticity of fees and commissions with respect to the policy rate is 0.93, which means that for each 1% decline in the policy rate, income from fees and commissions increases by 0.93%. And the longer that low interest rates persist, the more this rebalancing effect is reinforced.” This means that banks in order to move toward fee-based businesses may develop different frameworks of bank and FinTech collaboration to speed up innovation and time to market responsiveness. This is increasingly important, if we consider that as persistent low interest rates tend to reduce bank profits mainly by depressing interest margins, “banks adjust their activities in an effort to offset that reduction, at least partially. (…) And they reveal that funding tends to shift from short-term market funding toward deposits” (BIS, 2019b, p. 12).

On this outline, we underline that the construct of open banking—throughout the PSD2 directive and similar regulations around the world—is paving the way for third parties to work more and more on banks' deposits with true chances to develop mechanisms of further new disintermediation unless banks react actively in becoming good and better at innovating and offering new propositions to their customers.

This situation underlines the urgent need for banks to counteract fiercely.

While digitally native firms often have an edge on data skills, banks may retain an advantage in handling soft, context-dependent information that cannot be reliably tracked from quantitative metrics. Even if the importance of this factor varies considerably across bank business segments, it exists in many of them—including, for example, small business lending and advisory services. This is another interesting asset for banks to consider and leverage on by improving, overall, through partnering with FinTechs. In fact, 79 percent of leading banks have partnered with FinTechs to foster innovation (McKinsey, 2019, p. 34).

Now the challenge is to make these partnerships working at their best. All this goes toward the core mechanism of value co-creation, which has been mentioned above, that is, the integration of resources of several actors. If we take a service perspective, resources may regard people, systems, infrastructures, and information (e.g., Grönroos, 2006), and also knowledge and skills are becoming central resources for a company (Vargo and Lusch, 2004, 2008; Lusch and Nambisan, 2015; Omarini, 2015). All of them are important ingredients for a platform to develop its service innovation (Lusch and Nambisan, 2015) as this is going to be the new financial intermediation paradigm.

Moreover, banks also benefit from a fairly sticky customer base that is from switching deposit institutions and is likely to work with banks with which it has an existing relationship. Therefore, retaining and developing a loyal customer base will be increasingly important in the future (Omarini, 2013). While the relationship-based dimensions of banking may be on a long-term trend of erosion, due to changes in lending technology and banking regulation, they are unlikely to disappear altogether.

## Data Availability Statement

The original contributions generated for the study are included in the article/supplementary material, further inquiries can be directed to the corresponding author/s.

## Author Contributions

AO conceived the presented idea. She developed the study conception and design as well as the drafting of the manuscript and its critical revision.

## Conflict of Interest

The author declares that the research was conducted in the absence of any commercial or financial relationships that could be construed as a potential conflict of interest.
